# The Lone Star tick, *Amblyomma americanum*, salivary factors exacerbate the clinical outcome of Heartland virus disease in a small animal model

**DOI:** 10.1038/s41598-023-40397-x

**Published:** 2023-08-16

**Authors:** Erin S. Reynolds, Jacob T. Wooldridge, Heather L. Stevenson, Saravanan Thangamani

**Affiliations:** 1 SUNY Center for Vector-Borne Diseases, Upstate Medical University, Syracuse, NY USA; 2 Institute for Global Health and Translational Sciences, Upstate Medical University, Syracuse, NY USA; 3 Department of Microbiology and Immunology, Upstate Medical University, Syracuse, NY USA; 4 Department of Pathology, University of Arkansas for Medical Sciences, Little Rock, AR USA; 5 Department of Pathology, University of Texas Medical Branch at Galveston, Galveston, TX USA

**Keywords:** Microbiology, Pathogenesis

## Abstract

Heartland virus was first isolated in 2009 from two patients in Missouri and is transmitted by the Lone Star tick, *Amblyomma americanum.* To understand disease transmission and pathogenesis, it is necessary to develop an animal model which utilizes the natural route of transmission and manifests in a manner similar to documented human cases. Herein we describe our investigations on identifying A129 mice as the most appropriate small animal model for HRTV pathogenesis that mimics human clinical outcomes. We further investigated the impact of tick saliva in enhancing pathogen transmission and clinical outcomes. Our investigations revealed an increase in viral load in the groups of mice that received both virus and tick salivary gland extract (SGE). Spleens of all infected mice showed extramedullary hematopoiesis (EH), depleted white pulp, and absence of germinal centers. This observation mimics the splenomegaly observed in natural human cases. In the group that received both HRTV and tick SGE, the clinical outcome of HRTV infection was exacerbated compared to HRTV only infection. EH scores and the presence of viral antigens in spleen were higher in mice that received both HRTV and tick SGE. In conclusion, we have developed a small animal model that mimics natural human infection and also demonstrated the impact of tick salivary factors in exacerbating the HRTV infection.

## Introduction

**Figure 1:**
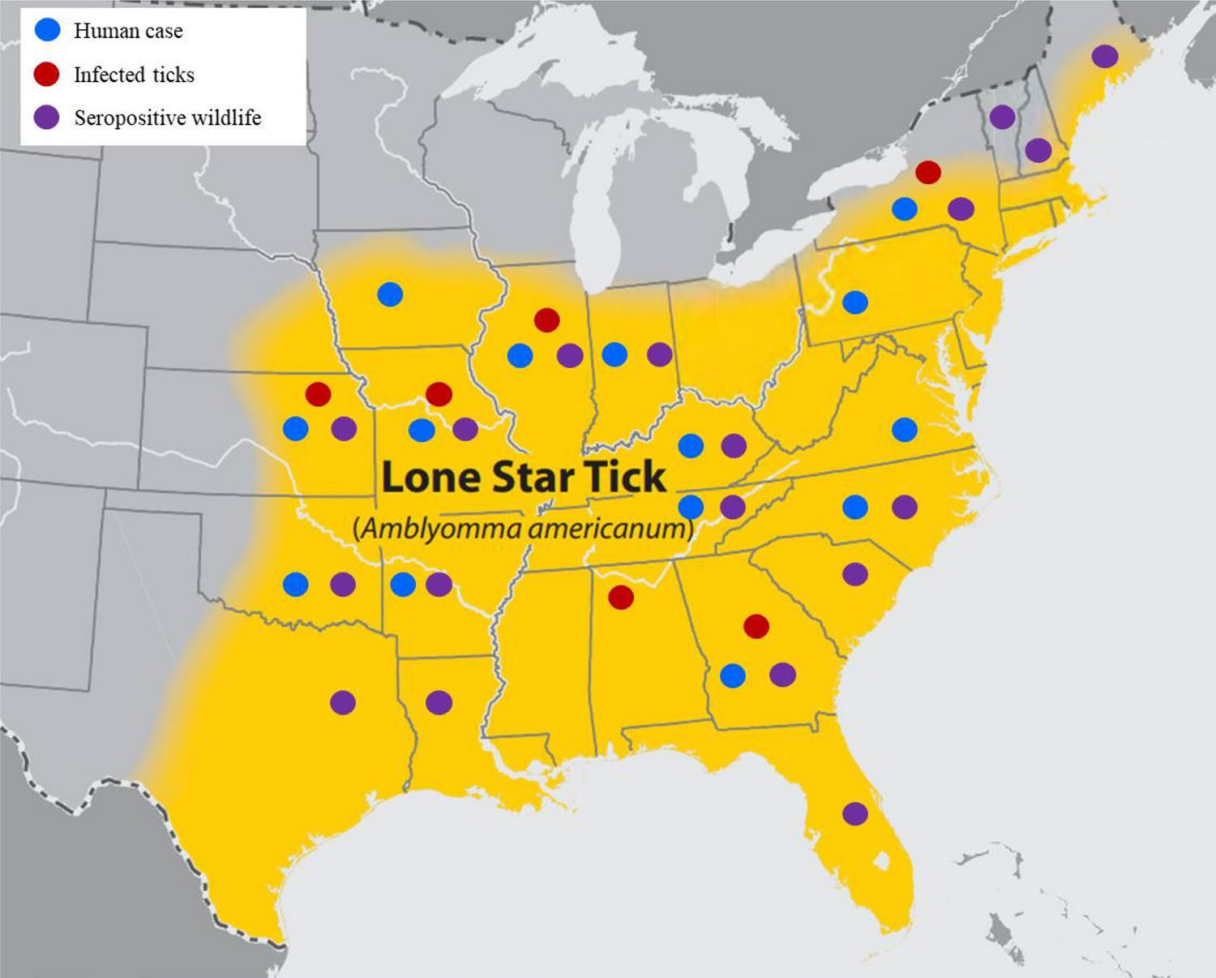
Known distribution of *Amblyomma americanum* and Heartland virus. Current CDC estimates for *A. americanum* distribution (https://www.cdc.gov/ticks/geographic_distribution.html) overlayed with known occurrence of human case (blue dot), infected ticks (red dot), and seropositive wildlife (purple dot).

Ticks have been a known vector for viruses that cause human disease for over a century^1^ and the geographic distribution of ticks and the pathogens they transmit spans much of the globe^2,3^. Although the reasons are not fully understood, the number of cases of tick-borne viruses are increasing over the last few decades^3–7^. Since 2007, newly discovered tick-borne viruses, including Dabie bandavirus, the causative agent of Severe Fever and Thrombocytopenia Syndrome (SFTS), Bourbon virus, the causative agent of Bourbon virus disease, and Heartland virus (HRTV), the causative agent of Heartland virus disease, have been identified by both human cases and retrospective examination of human or animal serum and field collected ticks^8–11^. Heartland virus (family *Phenuiviridae*, genus *Bandavirus*) is an emerging tick-borne disease, first isolated in 2009 from two ill humans in Missouri, USA^9^. Following the initial discovery of HRTV there have been over 50 confirmed human cases spanning 14 states (Fig. 1)^12,13^. Symptoms of HRTV disease are similar to the closely related SFTSV, which was isolated in China in 2007^8^, and include fever, headache, fatigue, nausea, muscle and joint pain, and anorexia, with blood work showing thrombocytopenia, leukopenia, and elevated liver enzymes^9,12,14^. There have been four known fatal cases of HRTV, all in individuals with substantial comorbidities^14–17^. Screening of blood collected in 2013 from blood donors in northwestern Missouri estimated human seroprevalence in endemic areas at 0.9%, while testing of individuals with disease of unknown etiology and symptoms consistent with HRTV disease identified acute and previous infection across seven states^18,19^. The Lone Star tick, *Amblyomma americanum*, has been implicated as the vector for HRTV. Pools of field collected *A. americanum* ticks caught adjacent to the homes of the index patients, as well as in nearby regions of Missouri and Kansas, have tested positive for HRTV^20–23^. Lone Star ticks are aggressive in host-seeking and readily feed on humans^24–26^. The current range of *A. americanum* includes much of the Southeast, Northeast, and Midwest United States but reports of geographic expansion, as well as modeling of climate change scenarios indicate increased areas where environmental conditions are suitable for the establishment of *A. americanum*^27–31^. Although human cases of HRTV have not been detected in all areas where *A. americanum* is present, limited surveillance of wild and domestic animals in these areas have demonstrated seropositivity in multiple species including white-tailed deer (*Odocoileus virginianus*), raccoons (*Procyon lotor*), coyotes (*Canis latrans*), and moose (*Alces alces*) in 18 states and HRTV infected ticks in six states (Fig. 1)^21,23,32–37^. Efforts to develop suitable animal models to study HRTV transmission and pathogenesis have been limited and immunocompetent animals do not recapitulate symptoms of human disease^38–40^. In both previously published work and based on our experiments only animals which lack Interferon (IFN) receptors appear to be susceptible to HRTV infection^38,41,42^. To date, animal model development efforts have focused primarily on the interactions between pathogen and host, without thoroughly examining the role the arthropod vector plays in disease transmission and pathogenesis. The feeding process of Ixodid ticks is very different from other blood-feeding arthropods in regards to both the amount of blood collected and the duration of the feeding. While the bite of a mosquito, flea, or midge can be completed within a few minutes, Ixodid ticks feed for several days or weeks and significantly increase in size to accommodate a large blood meal. To facilitate feeding and avoid detection tick saliva has evolved a complex combination of pharmacologically active compounds that assist in blocking, modulation, and suppression of host the itch and pain response, immune response, wound healing, and coagulation responses^43,44^. These compounds have been shown to change throughout the feeding process and may vary between tick life stages^45,46^. While the purpose of these compounds benefit the vector they also create an environment that is permissive to pathogen transmission through a process called saliva-activated transmission (SAT) and exacerbate disease course^43,47–49^. To understand the transmission and pathogenesis of arthropod-borne viruses it is essential to examine the interactions between pathogen, host, and vector. The overall aims of these studies were to identify a small animal model which manifested clinical and pathological changes consistent with human cases of HRTV infection and then evaluate the impact of tick saliva on pathogenesis.

## Materials and methods

### Ethics statement

Animal experiments were conducted in compliance with the guidelines of the Association for Assessment and Accreditation of Laboratory Animal Care International (AAALAC) in an AAALAC-approved facility. Study protocols (1504024 and 0112059) were approved by the University of Texas Medical Branch (UTMB) Institutional Animal Care and Use Committee (IACUC) Following arrival or transfer to UTMB Animal Biosafety Level 3 (ABSL3) facilities animals were allowed to acclimate to the facility prior to study initiation.

### Tick salivary gland extract

Adult *Amblyomma americanum* ticks which were free of known human pathogens were purchased from Oklahoma State University Department of Entomology were allowed to feed on New Zealand White rabbit. Approximately 2 weeks post attachment (13–15 days) but before completion of feeding one male and four female engorged ticks were removed. Salivary glands were dissected, pooled, and stored in 300 µL Phosphate Buffered Saline (PBS) (Corning) at – 80 °C. Prior to experimental study, the pooled salivary gland extract (SGE) was thawed over wet ice and homogenized in a TissueLyser II (Qiagen). Based on the total volume of the suspension each animal received 15 µL of SGE which was approximately half of one salivary gland.

### Virus

Heartland Virus strain MO-4, which was isolated from the index human case^9^, was provided by the World Reference Center for Emerging Viruses and Arboviruses (WRCEVA) (UTMB). Virus was passaged four times on Vero E6 cells (CRL-1586) (American Type Culture Collection). The virus titer was determined via focus-forming assay (FFA) adapted from previously published methods^50,51^. Vero E6 cells were seeded onto 12-well plates (Corning) and allowed to adhere overnight. A culture medium of Dulbecco’s Modified Eagle Medium (DMEM) (Gibco) supplemented with 2% Fetal Bovine Serum (Hyclone) and 1% Penicillin–Streptomycin (Corning) were supplied and plates were maintained at approximately 37 °C with 5% CO_2_. Immediately prior to infection, media was aspirated from the plates and serial dilutions of HRTV were dispersed onto the cell monolayers in triplicate. One column of wells was utilized as a negative control and had media dispensed onto it. Plates were incubated for 1 h and gently rocked every 10 to 15 min to ensure virus distribution. Following incubation 0.8% methylcellulose overlay was added and plates were maintained for 4 days. Methylcellulose was removed and plates were fixed with methanol:acetone (1:1). Plates were allowed to air dry completely. Cell membranes were permeabilized by incubating with 0.5% Triton X-100 (Sigma-Aldrich) in PBS for 5 min and then washed with 0.05% Tween20 (Sigma-Aldrich) in PBS (PBST). A blocking solution of PBST with 5% goat serum (Sigma-Aldrich) and 1% bovine serum albumin (Fisher Scientific) was incubated on wells for 1 h followed by an additional hour incubation of mouse immune ascitic fluid against HRTV strain MO-4 (1:500) (WRCEVA). Plates were washed with PBST prior to a 1-h incubation with goat anti-mouse IgG secondary antibody conjugated to horseradish peroxidase (1:1000) (Invitrogen). Following another PBST wash AEC substrate, prepared and used in accordance with the ImmPACT AEC Peroxidase (HRP) Substrate kit (Vector Labs), was added to each well and allowed to develop in the dark.

### Host susceptibility study

Initial experiments were conducted to assess the susceptibility of various strains of laboratory mice to HRTV infection by intraperitoneal (IP) and footpad (FP) injection under isoflurane anesthesia. All mice received a single injection of 1.49 × 10^5^ FFU HRTV and were monitored up to 18 days post-infection (dpi). Uninfected control groups were utilized for each strain of mouse infected with HRTV. Control mice were grouped in the number, sex, strain, and age as experimentally infected mice but received a single IP injection of PBS instead of HRTV. These mice were handled in the same manner and underwent the same procedures as experimentally infected mice. Immunocompetent CD-1 mice and C57BL/6J, purchased from Charles River Laboratories (Wilmington, MA) and The Jackson Laboratory (Bar Harbor, ME), respectively, were first assessed. Both strains of mice were approximately 7 weeks old at study initiation. Following the assessment of immunocompetent mouse strains, two IFN receptor knock-out strains of mice were evaluated. STAT1 knock out mice, which have deficient response to IFN-α/γ, were purchased from Taconic Biosciences (Germantown, NY) and A129 knock out mice, which have deficient response to IFN-α/β, were bred and maintained in the specific pathogen free colony facility at UTMB**.** STAT1 mice were approximately 5 weeks old at study initiation and A129 mice were 5 to 6 weeks old at study initiation.

Following infection all mice were monitored for clinical manifestation of disease (Supplementary Table S1), whole blood was collected by retro-orbital sinus prior to infection and every 2 to 3 days thereafter to evaluate viremia, and at study termination whole blood was collected by cardiac puncture and the spleen, liver, kidneys, and muscle adjacent to the injection site were collected to determine viral load. Mice were sedated by isoflurane inhalation for FP injection and retro-orbital sinus blood collection. Euthanasia was performed by CO_2_ inhalation followed by cervical dislocation. Blood and tissue samples were stored in either TRIzol LS or TRIzol (ThermoFisher Scientific) until homogenized for RNA extraction and subsequent quantitative rt-PCR analysis.

### Impact of tick saliva on HRTV pathogenesis

Eighteen A129 mice, 10 males and 8 females, were used to evaluate the effects of tick saliva on HRTV pathogenesis. Due to limited availability of mice at 3 weeks old, at study initiation three female mice aged 33 days and one female mouse aged 22 days were designated as uninfected controls. The remaining 10 male and four female mice, all aged 22 days, were weighed and designated to received HRTV only or HRTV + SGE. The mean weight for these groups was 9.32 g and 9.18 g, respectively. All mice received a single injection of 45 µL in the right hind footpad. Negative control mice received culture medium, the virus only mice received 1.35 × 10^5^ FFU HRTV, and virus plus SGE mice received 1.35 × 10^5^ FFU HRTV combined with SGE. Monitoring for clinical disease and blood sampling were completed as described above. Additional whole blood samples were collected at euthanasia for hematology and clinical chemistry analysis. Mice were euthanized at either 3 or 8 dpi and necropsy was immediately performed. Spleen, liver, kidneys, brain, heart, lungs, stomach, small intestine, and testes, where applicable, were subdivided and stored in TRIzol for RNA extraction or 10% Neutral Buffered Formalin (Fisher Scientific) for histopathological analysis.

### Hematology

Prior to infection and at euthanasia whole blood was collected into tubes containing K3 EDTA and gently inverted until mixed. Samples were analyzed on the date of collection by Hemavet 950FS instrument (Drew Scientific). The following parameters were evaluated: red and white blood cell counts; percent and counts of leukocytes; hemoglobin; hematocrit; mean corpuscular volume; mean corpuscular hemoglobin; mean corpuscular hemoglobin concentration; and platelets.

### Clinical chemistry

At euthanasia, whole blood was collected into serum separator tubes and analyzed on the date of collection. Samples were allowed to clot for at least 20 min before being centrifuged for 10 min at 1500×*g*. The analysis was completed by Vetscan VS2 instrument (Abaxis) utilizing Preventive Care Profile Plus Rotors. Parameters evaluated were blood urea nitrogen, creatinine, alanine aminotransferase, alkaline phosphatase, aspartate aminotransferase, total bilirubin, glucose, calcium, total protein, albumin, globulin, sodium, potassium, chloride, and total carbon dioxide.

### RNA extraction

Total RNA was extracted from tissue and whole blood samples by RNeasy Mini Kits (Qiagen, LLC) in accordance with previously published methods^52^ which were modified from the TRIzol Reagent User Guide and RNeasy Mini Handbook. Immediately following collection, tissue and whole blood samples were stored in TRIzol or TRIzol LS reagent, respectively. Samples were stored refrigerated overnight to allow pathogen inactivation prior to being transferred to − 80 °C until RNA extraction was completed. Prior to extraction tissues were homogenized by TissueLyser II (Qiagen). Whole blood samples were not homogenized. RNA quantity and purity were assessed by NanoDrop 1000 (Fisher Scientific) or DS-11 + (DeNovix) spectrophotometer.

### Pathogen detection and quantification by real-time polymerase chain reaction

Quantitative real-time polymerase chain reaction (qRT-PCR) was completed by combining the components of iTaq Universal Probes One-Step Kits (Bio-Rad) and HRTV specific primer probe^20^. This mix was dispensed onto iQ 96-Well PCR Plates (Bio-Rad) and up to one microgram of RNA was added. Plates were sealed and qRT-PCR was run on an iQ5 Real-Time PCR system (Bio-Rad) in accordance with the iTaq Universal Probes One-Step Kit protocol. To quantify viral load, RNA was extracted from two samples of known infectivity. The resulting RNA was serially diluted, in duplicate, and qRT-PCR was conducted with the same materials and protocols used for experimental samples. The resulting threshold cycle (C_T_) values were averaged for each dilution and plotted with the log of the viral concentration to generate a linear equation and determine the standard curve.

### Histopathology and immunohistochemistry

Tissues were formalin fixed in accordance with UTMB standard operating procedures for BSL-3 agents prior to transfer to BSL-2 laboratories. Tissues were paraffin embedded and sectioned at 5 µm thickness. Hematoxylin–eosin staining was completed on all organs collected and immunohistochemistry for HRTV antigen was completed for spleens adapted from previously published methods^53^. Slides were deparaffinized and rehydrated in xylenes and decreasing concentrations of ethanol. Slides were submerged in DAKO Target Retrieval Solution (Agilent) and microwave heated for 20 min. After cooling to room temperature slides were then incubated for 5 min while protected from light in 3% H_2_O_2_ to quench endogenous peroxidase. Mouse-On-Mouse kit (MOM) (Vector Labs) was utilized to reduce high background staining caused when mouse primary antibodies are used on mouse tissues. Slides were incubated for 1 h with MOM Mouse IgG Blocking Reagent. Mouse anti-HRTV monoclonal antibody (MAb) 2AG9^54^ diluted 1:250 in MOM diluent and incubated for overnight at 4 °C. Secondary antibody, MOM biotinylated anti-mouse IgG diluted 1:250 in MOM working solution, was incubated for 20 min at room temperature. Incubation with Streptavidin-peroxidase Ultrasensitive Polymer (Sigma-Aldrich) followed by ImmPACT AEC Peroxidase (HRP) kit (Vector Labs) were completed in accordance to vendor-supplied protocols to detect biotinylated antibody. Slides were counterstained with Harris hematoxylin (Fisher Scientific) and mounted in Vectamount AQ Aqueous Mounting Medium (Vector Labs).

### Statistics

Statistical analyses were performed using GraphPad Prism 9 (GraphPad). Data for each group was averaged and the standard error of the mean (SEM) calculated (95% CI). Statistical significance was determined for results which had a p-value < 0.05. Viremia and viral load data were evaluated using a two-tailed, unpaired t-test with Welch’s correction. Hematology data were evaluated using Brown-Forsythe and Welch’s ANOVA test with Dunnett’s T3 multiple comparisons test.

## Results

### Host susceptibility evaluation

**Figure 2:**
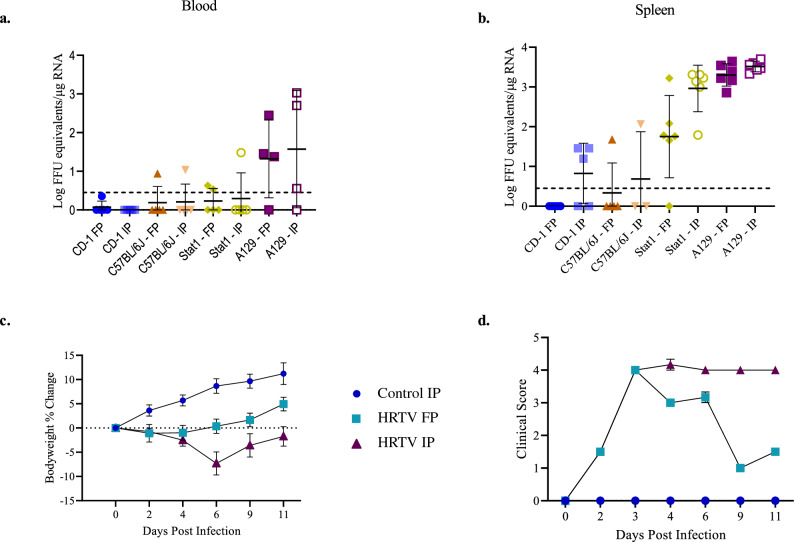
Viral RNA detection in blood and signs of disease in host susceptibility evaluation experiments. HRTV viral RNA detected by qRT-PCR in blood (**a**) and spleen (**b**) across mouse strains. Mice were exposed to HRTV via footpad (FP) or intraperitoneal (IP) injection. Bodyweight changes (**c**) and clinical score (**d**) of A129 mice following challenge with HRTV. Error bars show SEM.

The uninfected control mice utilized for each experiment did not develop any clinical symptoms, weight loss, or injection site complications. Immunocompetent CD-1 and C57Bl/6J mice were refractory to HRTV when infected by FP and IP injection. All mice used for these experiments were female. Five CD-1 mice were inoculated by each route. Five C57BL/6J mice were inoculated by each route, but two mice in the IP group did not recover from the anesthesia used during inoculation. Clinical observations and body weight measurements were performed following the infection, but no signs of illness or weight loss developed. Blood was collected at 2, 4, 6, 8, 10, 12, and 18 dpi. One CD-1 mouse which had been inoculated via the FP had detectable viral RNA at 10 and 18 dpi (Fig. 2). One C57BL/6J mouse which had been inoculated via the IP route had detectable viral RNA at 6 dpi while one C57BL/6J mouse inoculated via the FP had detectable viral RNA at 12 dpi (Fig. 2). Viral RNA was not detected in any other CD-1 or C57BL/6J mouse. At 18 dpi all surviving mice were euthanized via CO_2_ inhalation followed by cervical dislocation. Necropsy was performed on all mice to collect spleen, kidney, liver, and muscle adjacent to the injection site. There were no gross pathology observations. Organs were subdivided and placed in either Trizol or 10% formalin. Viral detection by qRT-PCR indicated viral RNA was present in three of the spleens for the IP infected CD-1 mice, one spleen for the IP infected C57BL/6J mice, and one spleen for the FP infected C57BL/6J mice (Fig. 2). The FP inoculated C57BL/6J mouse which was viremic at 12 dpi was the same mouse with virus detected in the spleen. No other mice had viral RNA detected in the spleen or blood.

Stat1 mice had increased susceptibility to HRTV as compared to immunocompetent mice. Groups of six female mice were injected with HRTV via the FP or IP route. No clinical symptoms of disease or weight loss was observed. Blood was collected on 2, 4, 7, 9, 11, and 18 dpi. At 2 dpi three IP injected mice and one FP injected mouse had detectable viral RNA (Fig. 2). One additional FP injected mouse exhibited viral RNA at 9 dpi. Necropsy was performed at 18 dpi and there were no gross pathology observations. Viral RNA was detected by qRT-PCR in the spleens of all IP injected Stat1 mice and all but one spleen of FP injected mice (Fig. 2). Viral RNA was also detected in the liver of one IP injected mouse which also had viral RNA detected in blood at 2 dpi.

A129 mice were susceptible to HRTV when injected via the FP and IP route. Groups of six mice (three males and three females per group) were inoculated on day 0 and monitored until 11 dpi. Clinical symptoms developed at 2 dpi and peaked between 3 and 4 dpi for both infected groups (Fig. 2). Initially mice presented with reduced grooming and progressed to a dull or rough coat and decreased cage activity that was normal when provoked. Beginning at 4 dpi the clinical symptoms of the FP infected mice began to decrease in severity, but mice did not return to normal. Clinical symptoms of the IP infected mice did not increase or decrease in severity after 4 dpi.

At 2 dpi infected mice began to have weight loss (Fig. 2). This was not consistent between routes of infection or within groups. Due to the natural differences in weight ranges between mice, weight changes were evaluated by comparing the percent change for each animal from their day 0 baseline weight. The FP infected mice had a peak weight loss at 2 dpi with an average loss of 1.1%, individual weight changes ranged from a 7.5% loss to a 5.1% gain. By 6 dpi the group average weight change was greater than baseline weights. Outside of the one 7.5% weight loss at 2 dpi no other measurement reflected a ≥ 5% weight loss and two of the three male mice in this group did not have any weight loss. Weight loss for the IP infected mice peaked at 6 dpi with an average loss of 7.3%, individual weight changes ranged from a 15.7% loss to a 2.2% gain. All mice in this group exhibited weight loss during the experiment and although one male mouse had a maximum weight loss of 1.7% all other mice in the group had a loss that was > 5%. By 11 dpi all mice but one female had begun gaining weight. Of the five mice that were gaining weight only two surpassed their baseline values.

Blood was collected at 2, 4, 6, and 11 dpi and tested for viral RNA by qRT-PCR. One female mouse in the FP infection group did not have viral RNA detected in the blood. Viral RNA was detected in all other infected mice during at least one collection between 2 and 6 dpi (Fig. 2). By 11 dpi no viral RNA was present. At necropsy the spleen, kidney, liver, and muscle adjacent to the injection site were collected to viral load analysis and histopathology. Viral RNA was found in spleens from all infected mice by qRT-PCR.

Five-week old Stat1 mice did not develop clinical signs of illness but had viral RNA present in the spleens and blood of most animals that were infected. Tick saliva exacerbates Heartland virus clinical disease in A129 mice.

**Figure 3:**
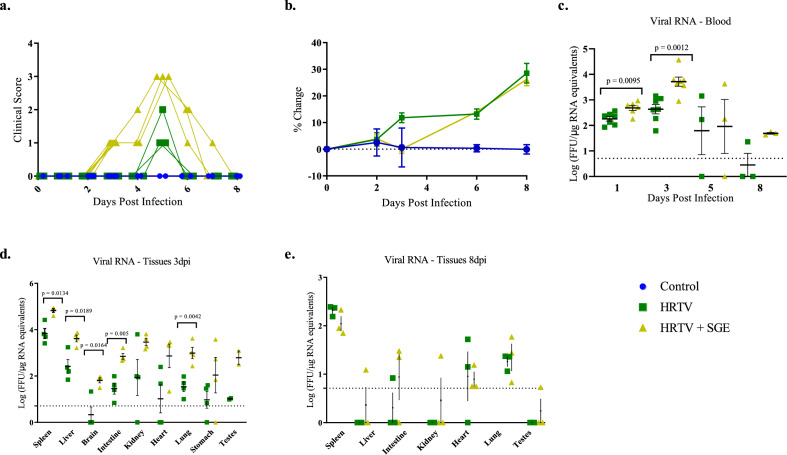
Clinical signs of disease and viral RNA detection in impact of tick saliva on HRTV pathogenesis experiment. Animals were observed and weighed daily to assess disease and assigned a cumulative score based on the scoring table in Table S1. Individual clinical scores and body weight changes from baseline are shown (**a**,**b**). Blood was collected at intervals post infection and tissues were collected at termination to determine viral load by qRT-PCR (**c**–**e**). Viral load data are expressed as FFU equivalents per microgram of RNA following normalization to a standard curve. Statistical significance between infected groups was determined using an unpaired two-tailed t-test with Welch’s correction. Error bars show SEM.

All mice received a single injection of 45 µL in the right hind FP. Uninfected control mice did not develop any reactions at the injection site (Fig. 3.) Two mice which received HRTV only developed slight swelling of the hind foot on 1 dpi which expanded to include all mice between 3 and 4 dpi. Swelling increased between 5 and 6 dpi with one mouse having a reduced usage of the right hind limb. These reactions began to resolve at 7 dpi with only one mouse continuing to have slight swelling until study termination at 8 dpi. In contrast to the HRTV only group, at 1 dpi mice which received HRTV + SGE had bruising and moderate swelling of the hind foot. By 3 dpi the swelling had progressed to include the right hind leg and all mice had reduced usage of the effected limb. Swelling had not reduced by study termination, but two mice did regain full usage of the right hind limb. At 3 dpi mice in the HRTV + SGE group began to have reduced grooming and clinical signs of disease peaked for this group at 5 dpi when presented with dull or rough coat and reduced activity that became normal when provoked. Symptoms resolved for these animals between 7 and 8 dpi. Clinical symptoms were only observed in the HRTV only group at 5 dpi where mice had reduced grooming. The injection site reactions and clinical symptoms were more severe in the HRTV + SGE animals as compared to the HRTV only animals. Additionally, the onset of symptoms occurred earlier and had a longer duration in the HRTV + SGE animals.

At 1, 3, 5, and 8 dpi blood was collected to assess the presence of viral RNA. All infected mice had detectable viral RNA in the blood and this was significantly higher in the HRTV + SGE group than the HRTV only group at 1 dpi (p = 0.095) and 3 dpi (p = 0.0012) (Fig. 3). Viral RNA persisted longer in the blood of HRTV + SGE mice. At 8 dpi one of three HRTV only mice still had detectable viral RNA but three of three HRTV + SGE mice had detectable viral RNA. Mice in the HRTV + SGE group also had higher viral loads in the spleen (p = 0.0134), liver (p = 0.0189), brain (p = 0.0164), intestine (p = 0.005), lung (p = 0.042), and testes at 3 dpi than HRTV only mice (Fig. 3). Statistical significance for the testes could not be determined due to small sample size from each group (n = 2). Unlike in the host susceptibility experiments with A129 mice, splenomegaly was observed in all infected mice at both necropsy timepoints. This may have been due to younger mice being used for experiments utilizing tick saliva. Organ weights were not collected but based on observations and photos taken during necropsy, as well as slides of the spleens, splenomegaly was less severe in the HRTV only group.

**Figure 4:**
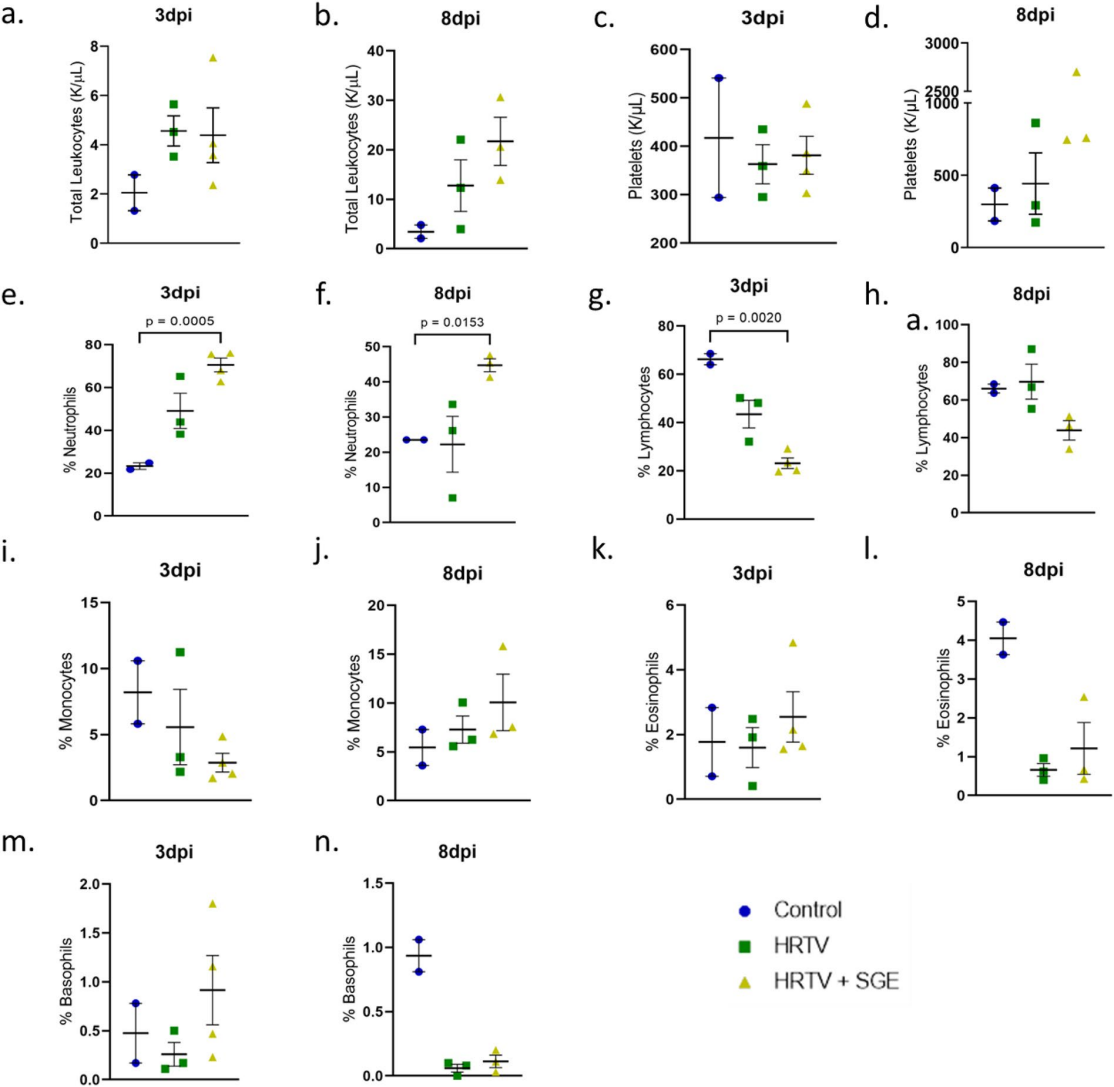
Hematology Hematology parameters were evaluated on whole blood collected at 3dpi and 8dpi terminations. Parameters evaluated included total leukocytes (**a**,**b**), platelets (**c**,**d**), and percentages of neutrophils (**e**,**f**), lymphocytes (**g**,**h**), monocytes (**i**,**j**), eosinophils (**k**,**l**), and basophils (**m**,**n**). Statistical analysis was performed using Brown-Forsythe and Welch’s ANOVA test with Dunnett’s T3 multiple comparisons test. Error bars show SEM.

Blood collected prior to infection and at necropsy for hematology analysis showed drastic changes from baseline levels at both 3 and 8 dpi for both infected groups (Fig. 4). Platelets counts remained stable between baseline and 3 dpi measurements and were increasing in the HRTV + SGE group at 8dpi (Fig. 4, Supplementary Table S2). Both infected groups of mice had white blood cell counts that were mildly elevated at 3 dpi and much more elevated at 8 dpi. This was more pronounced in the HRTV + SGE group. Despite the increased white blood cell counts, both infected groups had depleted lymphocytes at 3 dpi. The HRTV + SGE group was significantly reduced in comparison to the control mice (p = 0.0020) and these values had not returned to baseline levels at 8 dpi (Fig. 4). Both infected groups demonstrated increased neutrophils following infection with greater increases in the HRTV + SGE group at both 3 dpi (p = 0.0005) and 8 dpi (p = 0.0153) (Fig. 4) compared to the control mice. Blood collected at necropsy for clinical chemistry was inconclusive (Supplementary Table S3).

### Pathological examination of H&E stained liver and spleen sections and spleen sections stained for viral antigen by IHC

**Figure 5:**
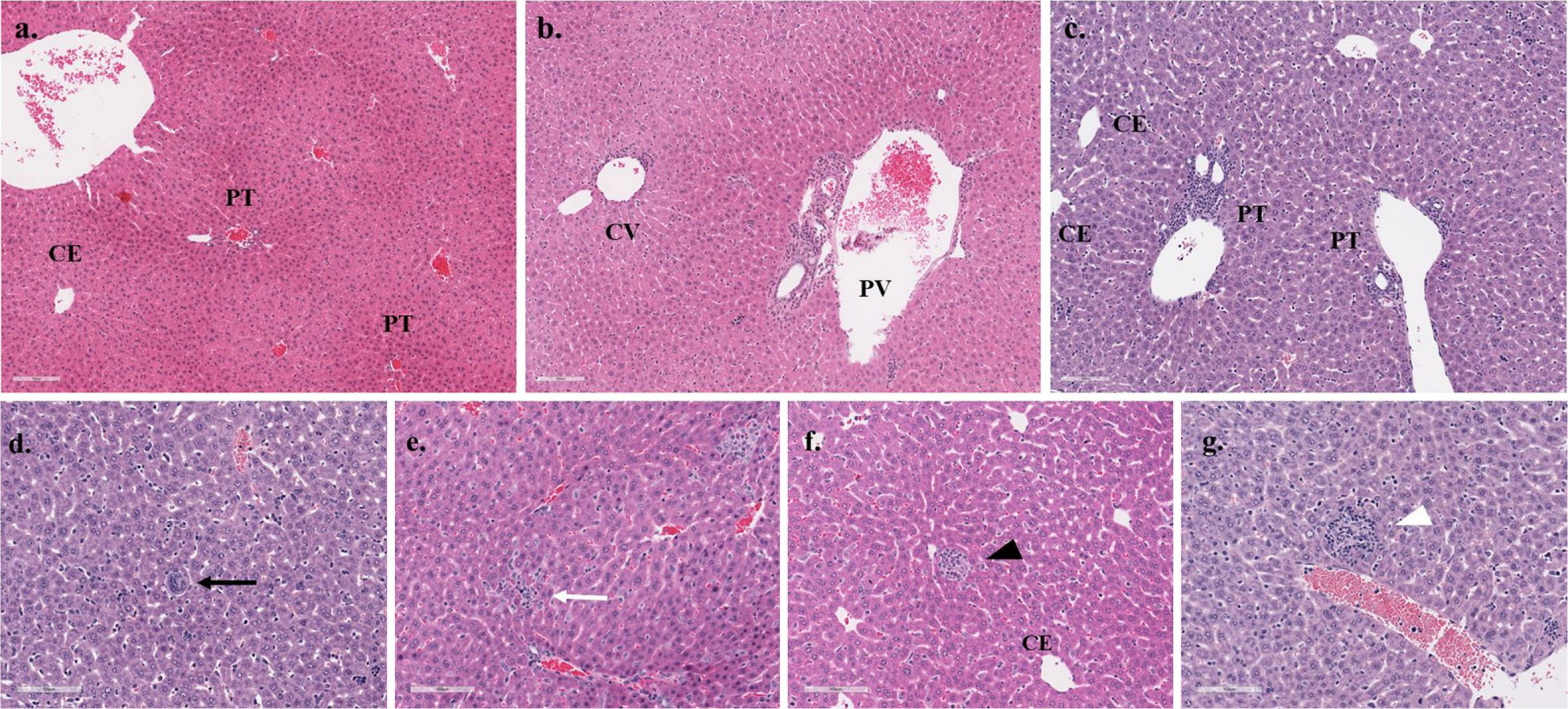
Liver pathology Hematoxylin and eosin staining of liver sections showed several histopathologic changes in infected mice. Control histology from an uninfected mouse with portal tract (PT) and central vein (CE) shown (**a**). Pathology from infected mice included portal vein inflammation with portal and central venulitis (PV, CV) (**b**), neutrophilic inflammation of the portal tract (**c**), lobular megakaryocytes (black arrow, **d**), lobular inflammation (white arrow, **e**), and granuloma-like lobular lesions with eosinophilic infiltrates (black arrowhead, **f** and white arrowhead, **g**). Complete pathology observations are available in S4 Table.

**Figure 6:**
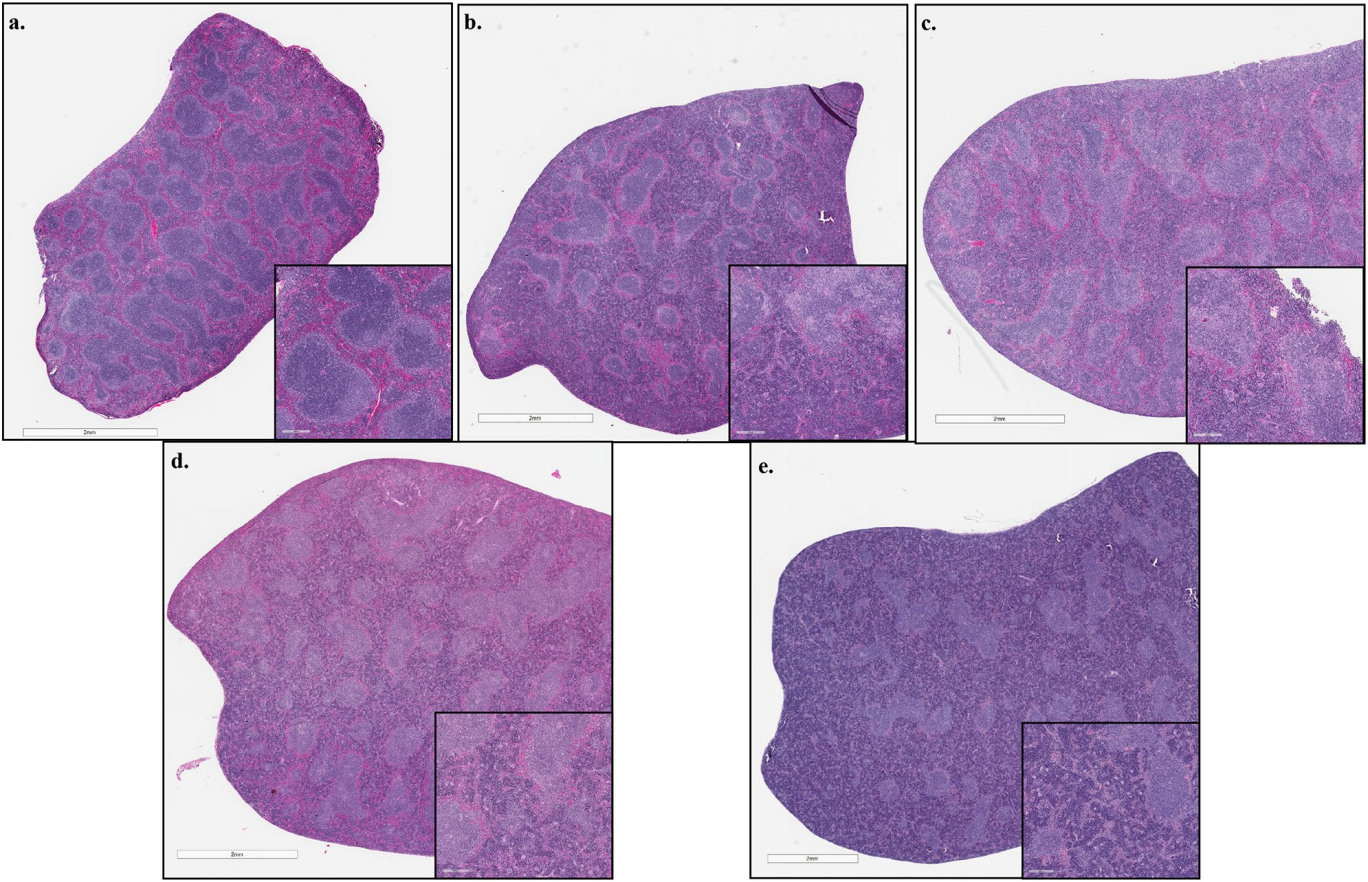
Spleen pathology Hematoxylin and eosin staining of spleen sections showed several histopathologic changes in infected mice. Control histology from an uninfected mouse (**a**) with normal structure. Reduction in average PALS diameter measured in HRTV (**b**) and HRTV + SGE (**c**) infected mice at 3 dpi. Increased hematopoiesis observed in spleens of HRTV + SGE mice at 3 dpi (**c**), HRTV mice at 8 dpi (**d**), and HRTV + SGE (**e**) mice at 8 dpi. Depleted white pulp and absence of germinal centers (**b**–**e**) in infected groups at 3 dpi and 8 dpi. Complete pathology observations and PALS averages for all mice located in S5 Table.

**Figure 7:**
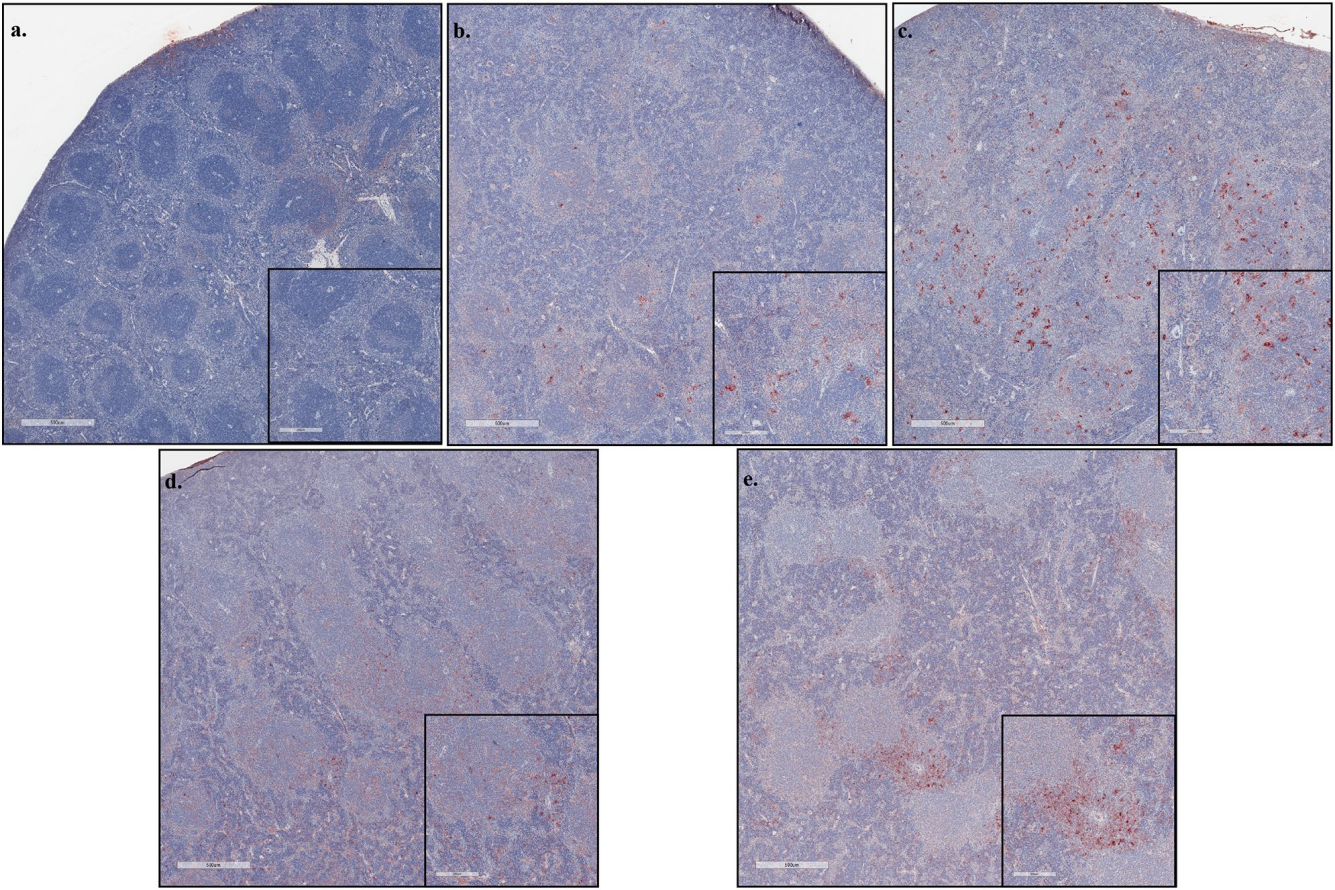
HRTV antigen detection in spleens Immunohistochemistry (IHC) was performed on spleen sections to visualize HRTV antigen (red signal) and counterstained with hematoxylin. Representative images of uninfected spleen with minimal non-specific staining (**a**), HRTV infected mouse at 3 dpi (**b**), HRTV + SGE infected mouse at 3 dpi (**c**), HRTV infected mouse at 8dpi (**d**), and HRTV + SGE infected mouse at 8 dpi (**e**). Scoring for antigen severity for all animals located in S5 Table. Scale bars are 500 µm for panels (**a**–**e**) and 200 µm for inset images.

The livers exhibited mixed inflammation of the portal tract and central vein present at 8 dpi and which was more severe in the HRTV + SGE group (Fig. 5, Supplementary Table S4). Megakaryocytes were observed at 3 and 8 dpi but were more prevalent in HRTV + SGE mice. Increased cellularity in sinusoidal spaces were observed in both groups at both timepoints but the cell phenotype could not be determined. Granuloma-like lobular lesions with eosinophilic infiltrates were more prevalent at 3 dpi and in the HRTV only mice. Spleens of all infected mice had extramedullary hematopoiesis (EH), depleted white pulp, and germinal centers were absent (Fig. 6, Supplementary Table S5). EH scores were slightly higher in the HRTV + SGE group at 3 dpi but were not different at 8 dpi. Both infected groups had slightly decreased average periarteriolar lymphoid sheath (PALS) diameter at 3 dpi. Uninfected control mice had an average PALS score of 307.79 (± 20.59) while HRTV only mice were 280.75 (± 46.06) and HRTV + SGE mice were 282.98 (± 20.26). Viral antigen was most prevalent in the HRTV + SGE group at 3 dpi and was similar between infected groups at 8 dpi (Fig. 7, Supplementary Table S5). This was consistent with the viral load analysis for the spleens.

## Discussion

Host susceptibility studies conducted here and previously described have demonstrated that many species of immunocompetent animals, including CD-1 and C57Bl/6 mice, hamsters, rabbits, chickens, goats, and raccoons, are refractory to HRTV infection^38^. Mice and hamsters with interferon receptor deficiency have been shown to have varying degrees of susceptibility to HRTV^38–42^. Stat2 hamsters aged 4 to 5 weeks and A129 mice aged 5 to 6 weeks, both IFN-α/β receptor deficient, have been shown to manifest clinical symptoms of disease, develop viremia, and viral RNA has been isolated in organs. Mortality occurred a small percentage of Stat2 hamsters^41^. Three week old IFN-α/β/γ receptor deficient AG129 mice were highly susceptible to HRTV infection and have been shown to develop a severe clinical disease similar to fatal human cases^38^. Despite the similarity to severe human cases, the calculated 50% lethal dose (LD50) in AG129 mice was 9 plaque-forming units (PFU)^38^. Seven to 11 week old IFNAR^−/−^ mice developed severe clinical disease in a dose dependent manner when injected subcutaneously, intraperitoneally, or intravenously^42^. Based on the low calculated LD50 for AG129 mice and the results from experiments utilizing IFN-α/β receptor deficient animals it was determined that 3-week old A129 mice would be the optimal model to investigate salivary factors of the arthropod vector, *A. americanum*, to exacerbate HRTV disease. In this work we have shown that A129 mice develop a non-lethal disease course with similarities to human cases of HRTV including splenomegaly, infection of multiple organs, and pathological changes in the liver and spleen.

Human cases of HRTV are often characterized by thrombocytopenia and leukopenia^9,14,16^ but these were not observed in A129 mice. This may be due to differences in the times post exposure when sampling occurs. For our work these parameters were not evaluated after 8 dpi while an exposed human may not know when they were exposed or seek medical attention until they have been ill for several days.

Furthermore, when HRTV is combined with *A. americanum* SGE the disease severity and duration increase compared to infection with virus alone. Tick saliva contains a repertoire of pharmacologically active protein and non-protein factors that are known to facilitate blood feeding and pathogen transmission. Tick salivary factors create an environment permissive to pathogen transmission and enhance severity via SAT^43,47–49,52^. Our result highlights the need to consider interactions between pathogen, host, and vector during the development of animal models for arboviruses as the vector feeding process has been shown to create an environment permissive to disease transmission and enhance severity via SAT^43,47–49,52^. Identification and characterization of salivary factors enhancing HRTV transmission will allow us to better understand the biology of HRTV transmission. The animal model described here will be useful in determining the specific components of tick saliva which impact the course of the disease. Isolating these components could provide potential targets for the development of vaccines to block disease transmission or potentially stop tick bites by preventing feeding.

As several tick species are able to transmit multiple pathogens a vaccine which would prevent successful feeding has the potential to be much more effective than developing vaccines for individual pathogens but may be much more difficult to design. The composition of tick saliva has been shown to vary between tick species and life stage as well as change throughout the feeding process and in response to the host on which the tick is feeding^43–46,48,49,55,56^. Identification of a single salivary factor across multiple species and life stages of tick may not be possible. The changing composition of tick saliva throughout the feeding process presents an additional challenge to vaccine development. The duration of attachment required for *Ixodes scapularis* to transmit various pathogens has been fairly well studied with some pathogens being transmitted as quickly as 15 min post attachment while other pathogens could take 24 to 48 h^57–59^. The minimum tick attachment time required for other tick species and pathogens, including *A. americanum* and HRTV, has been poorly studied and would require additional experiments to identify salivary factors which are presented early enough in the tick feeding process to prevent disease transmission.

### Supplementary Information

Supplementary Tables.
